# Effect of Hygrothermal Treatment on the Porous Structure and Nanomechanics of Moso Bamboo

**DOI:** 10.1038/s41598-020-63524-4

**Published:** 2020-04-16

**Authors:** Cuiyin Ye, Yanhui Huang, Qiming Feng, Benhua Fei

**Affiliations:** 1 Key Laboratory of Wooden Material Science and Application, Ministry of Education, Beijing Forestry University, Haidian, Beijing, 100083 PR China; 2 International Center for Bamboo and Rattan, Beijing, 100102 PR China

**Keywords:** Chemical modification, Biochemistry, Chemical biology, Plant sciences

## Abstract

Hygrothermal treatment is an environmentally friendly and efficient modification method. In this study, Moso bamboo was modified with hygrothermal treatments, and the results of nitrogen adsorption, X-ray diffraction (XRD), scanning electron microscopy (SEM) and nano indentation (NI) were then examined. Interestingly, the samples that underwent hygrothermal treatment at 180 °C and 117% RH (relative humidity) had the highest crystallinity (36.92%), which was 11.07% statistically larger than that of the control samples. Simultaneously, the total pore volume and average pore diameter (2.72 nm) dramatically decreased by 38.2% and 43.7%, respectively. The NI elasticity and hardness of the samples also reached the highest values under this condition; both increased by nearly 21% as compared with the control samples. Therefore, 180 °C is a favorable hygrothermal treatment temperature for Moso bamboo modification due to the porosity changes and the improvement of the nanomechanics of the cell walls.

## Introduction

Moso bamboo is a renewable and abundant material that is also biodegradable and environmentally friendly^1^. In the past few decades, more attention has been paid to the development of bamboo products as sustainable, cost-effective, and ecologically responsible alternative structural materials^2^. For example, bamboo weaving, bamboo scrimber, and laminated bamboo can be used in outdoor flooring, landscaping, and structural applications^3–5^. However, Moso bamboo, which is characterized by large amounts of hydrophilic hydroxyl groups and limited crystallinity, is unsatisfactory for engineered materials, which require a high dimensional stability and good mechanical properties. Therefore, modifications will be better to improve the dimensional stability and mechanical strength of Moso bamboo^6–8^.

Hygrothermal treatment is thought to be one of the most efficient methods of modification with minimal environmental hazards^9^. Studies of the three polymeric components (cellulose, hemicellulose, and lignin) after the heated or superheated steam treatment of woods have already been published^10–12^. Processing by superheated steam leads to the pyrolysis of these polymeric components^13^. According to previous studies, hemicellulose, as the most reactive biomass component, will be hydrolyzed into oligomeric and monomeric structures depending on the temperature^14–16^. During superheated steam processing, carbonic acids, mainly acetic acid, might initially be formed due to the cleavage of the acetyl groups of particular hemicelluloses^17–19^, leading to autocatalytic reactions of the cell wall constituents and an increase of relative crystallinity^13,20^. Lignin is the least reactive woody component, but high-temperature conditions will increase the reactivity of the lignin and the bonds within the lignin complex will be cleaved, causing autocondensation^19^. Moreover, it has been suggested that the hydroxyl groups in the microfibrils first degrade in amorphous regions during the hygrothermal process. There is a clear correlation between the changes in the weight and dimensional stability of biomass materials and the decrease of hydroxyl groups in the microfibrils. The reduction of free hydroxyl groups and the decrease of the hygroscopicity of bamboo during this period consequently improve its dimensional stability and durability^21,22^.

The changes in the mechanical properties of bamboo after thermal treatment have been recently studied. When heat-treated at 100–140 °C, researches proved a slight increase in the MOE of bamboo by 3.8–8.8% as compared to the control bamboo samples^23^. Another study demonstrated that 180 °C thermal treatment did not change the elastic modulus of the bamboo fiber, but the hardness showed increasing tendencies^24^. Others researchers found that heat treatment at temperatures above 200 °C resulted in a nearly 20.1% reduction in the MOE of bamboo samples^25^. The bamboo became brittle after treatment at high temperatures due to the additional degradation of chemical components.

However, the study of the effects of hygrothermal treatment on bamboo cell walls, especially on their porous structures and nanomechanics, is very limited^26,27^. In this study, saturated, superheated steam was used to modify bamboo. The treatment temperature was relatively low, which not only saves energy, but also avoids weakening the mechanical strength due to the degradation of cellulose at high temperatures. The mechanical properties and dimensional stability were mainly determined by the chemical components and porous structure of bamboo. The changes that occurred in the micro- and nano-scale structures due to hygrothermal treatment were explored via the nitrogen adsorption method, XRD, and SEM. Furthermore, to better understand the possible changes on the mechanical properties of bamboo, NI testing was applied for the determination of the quasi-static nanomechanical properties^28,29^. The objectives of this research are to reveal the inherent physical mechanism of dimensional stability improvement and the mechanical changes of bamboo caused by hygrothermal modification. Additionally, this research aims to determine the best treatment condition for modification to expand the application of bamboo products for sustainable and environmentally friendly buildings, as well as to prolong their service life.

## Materials and Methods

### Materials

Four-year-old Moso bamboo (*Phyllostachys edulis* (Carr.) H. De Lehaie) without stress (the samples were slowly dried), biological degradation, discoloration, knots, or other defects was selected from a bamboo plantation located in Yibin City, Sichuan Province, China. All experimental specimens were air-dried to a moisture content of 12% before further processing.

**Figure 1:**
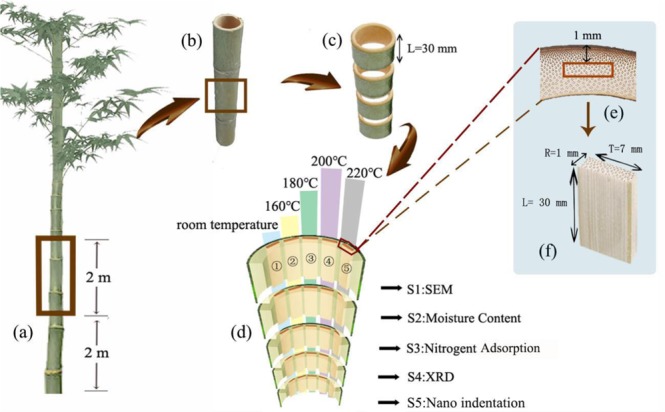
Schematic illustration of the preparation of the Moso bamboo samples.

**Figure 2:**
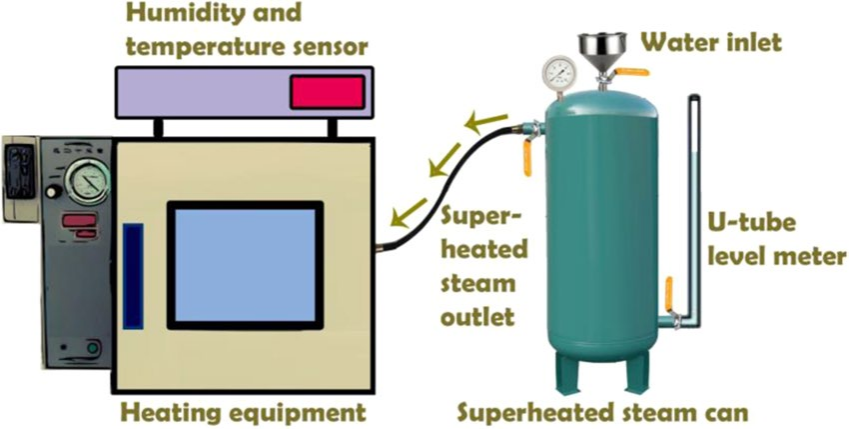
Self-developed, high-temperature, superheated steam device.

As can be seen from the schematic illustration in Fig. 1, the Moso bamboo was cut into 2-m-long units, and the unit that was 2 m away from the ground was chosen (Fig. 1(a–b)). Subsequently, the units were cut into 30-mm-long cylinders, then divided into sticks (1 mm away from the bamboo skin) with a final size of 30 mm (longitudinal) × 7 mm (tangential) × 1 mm (radial) (Fig. 1(c–f)). The sticks were modified by hygrothermal treatments in a self-developed, high-temperature, superheated steam device (Fig. 2). These bamboo samples were respectively treated at 160 °C, 180 °C, 200 °C, and 220 °C in a 117% RH environment for 1.5 h. The treated samples were cooled down and then stored in a desiccator.

To reduce the impact of the large variability in biological samples, the Moso bamboo was sawed into slices as illustrated in Fig. 1(d). The S1 slices were tested using a scanning electron microscope (SEM). The S2 slices were chosen and measured to extract the moisture contents of the samples. The S3 slices were cut into dimensions of 10 mm (L) × 4 mm (T) × 1 mm (R) for nitrogen adsorption measurement, while the S4 slices were milled into powder for X-ray diffraction (XRD) analysis. Finally, the S5 slices were investigated by NI.

### Nitrogen adsorption measurement

The slices were dehydrated with ethanol (30%, 60%, 90%, 95%, and 100% concentration). The samples were then dried using a supercritical extractor (SFE-2, USA). The CO_2_ in the thermostated drying chamber was delivered by a high-pressure pump. The operating temperature was set at 38 °C, and the working pressure was 1200 PSI. During the process, carbon dioxide could transform from liquid into supercritical fluid. The samples were dried for 3 h at a controlled flow of 10 L/min. In this way, the morphologies of the pores could be well preserved, and stress pressure on pores was also avoided during supercritical drying.

The nitrogen adsorption-desorption isotherms and specific surface areas were determined using a surface area and pore size analyzer (Autosorb iQ2-MP, USA) at 77 K. The samples were placed in the measuring cell at 80 °C for at least 10 h to remove all moisture or adsorbed contaminants. The samples were subsequently placed in an insulated tank filled with liquid nitrogen at a temperature of 77 K, where the pores of samples were filled with nitrogen molecules. The experimental data were then evaluated by the Brunauer-Emmett-Teller (BET) theory^30^ and the Barrett-Joyner-Halenda (BJH) theory^31^.

### X-ray diffraction (XRD)

The crystallinity and radial variation of the microfibril angle (MFA) of the bamboo fibers were examined with an X-ray diffractometer (Bruker D8 ADVANCE, USA) with CuKα radiation (*λ* = 1.54178 Å) at 40 kV and 40 mA. All XRD measurements were conducted via the reflection technique. 2θ, referring to the angle between the incoming and the scattered X-ray beam, was set from 5° to 45° at a scan rate of 2° min^−1^. The crystallinity of the cellulose in the bamboo cell wall (200 peak) was calculated using the following Segal method^32^:

$$CrI=\frac{{I}_{200}-{I}_{am}}{{I}_{200}}\times 100 \% ,(200)$$

where *C*_*r*_*I* is the crystallinity index (%), *I*_200_ is the maximum intensity of the (200) lattice diffraction angle (2θ = 22.1°), and *I*_*am*_ is the scattering intensity of the amorphous region (2θ = 18°). *I*_200_ and *I*_*am*_ have the same units.

The cellulose microfibril angle (MFA) was also measured via XRD with a symmetrical transmission mode. Each specimen was rotated around its normal axis with a rotation angle from 0° to 360° at a rotation speed of 1.5° per minute in a position of 2θ = 22.4°: the diffraction angle of the (200) plane of cellulose *I*_β_^33^. Subsequently, diffraction curves were fitted by GaussAmp bimodal functions (1), and the average MFA was then calculated by utilizing the well-established 0.6 T method given by Eqs. (2) and (3)^34,35^:

1 $$y={y}_{0}+{A}_{1}{e}^{\frac{-{(x-{x}_{c})}^{2}}{2{w}_{1}^{2}}}+{A}_{2}{e}^{\frac{-{(x-{x}_{c}-180)}^{2}}{2{w}_{2}^{2}}},$$

2 $$\overline{MFA}=\frac{({w}_{1}+{w}_{2})}{2}\times 1.2,$$

3 $$T={w}_{1}+{w}_{2},$$

where *y*_0_ is a constant, *x*_*c*_ and *x*_*c*_ - 180 correspond to the peak of the phi angle, *A*_1_ and *A*_2_ are the peak heights, *w*_1_ and *w*_2_ are standard deviations, $\overline{{\rm{MFA}}}$ is the average MFA, and *T* is the “angle T” as calculated from the diffraction pattern of the (200) plane.

### Scanning electron microscopy (SEM)

To obtain a better understanding of the effects of hygrothermal treatment on the properties of Moso bamboo, especially the morphological changes of porous structures, the S1 samples were examined with an environmental scanning electron microscope (ESEM, XL30ESEM-FEG)^36^. The working voltage was 5 kV in high vacuum mode. All the specimens were fastened to the mounting stubs using conductive carbon adhesive and were then sputter-coated with a layer of gold prior to examination.

### Nanoindentation (NI)

**Figure 3:**
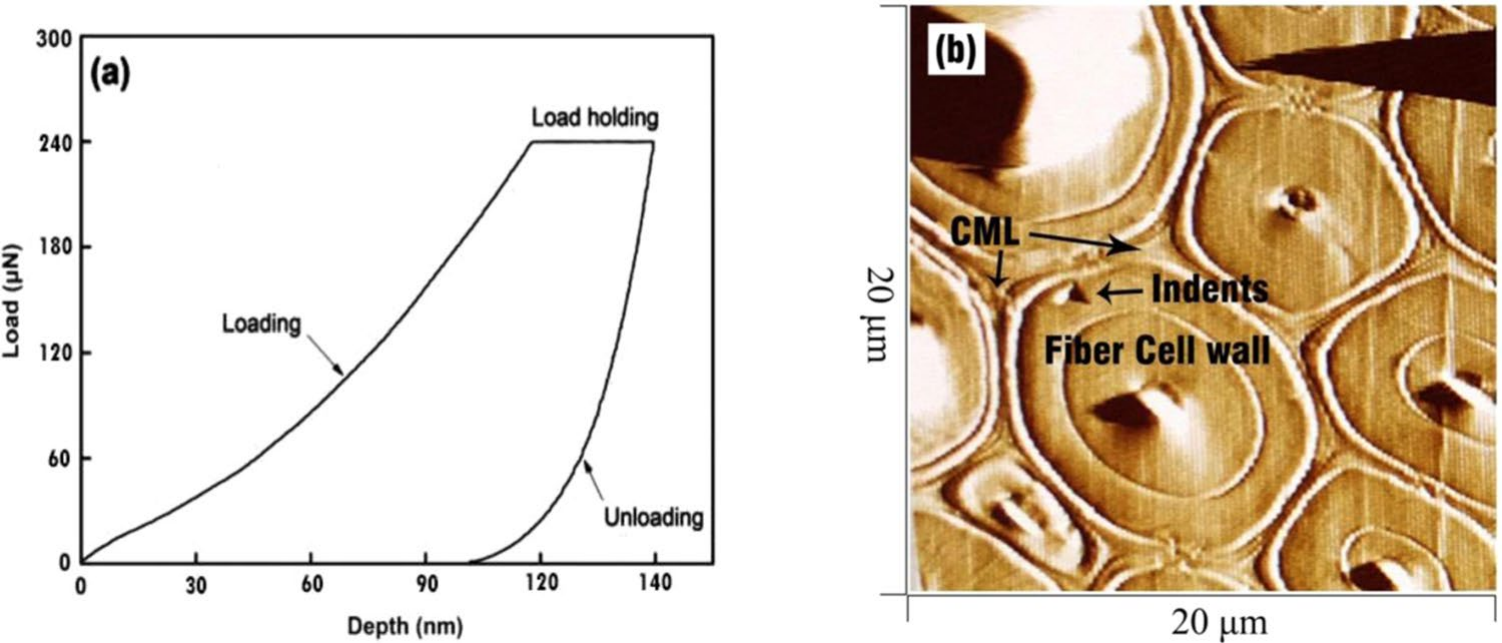
(**a**) Load-displacement curves and (**b**) indentation image; CML = cell middle lamella.

The dimensions of the samples for nanoindentation analysis were 10 mm (L) × 7 mm (T) × 1 mm (R). The bamboo samples were cut into a pyramid shape at the apex, and a smooth surface was created using a diamond knife. The bamboo samples were adhered to metal stubs with fast-curing adhesive. A nanoindenter (Triboindenter, Hysitron Inc., USA) was used to conduct nanoindentation tests due to its unique *in-situ* imaging function. Indentation was loaded over 5 s to a peak force of 250 μN, the maximum force was held for 6 s, and then unloading occurred over 3 s (Fig. 3). The final data represent an average of at least 20 indents on the cell walls, which contained four or five cells for each point.

According to a study by Yu *et al*. (2007), the elastic modulus (*E*) and hardness of materials can be calculated from the load-displacement curves of NI^37^. The elastic contact stiffness (*S*) can be determined according to the initial slope of the unloading curve (70–90%)^29^. The reduced elastic modulus *E*_*r*_ can also be subsequently computed:

4 $$\frac{1}{{E}_{r}}=\frac{1-{v}^{2}}{E}+\frac{1-{v}_{i}^{2}}{{E}_{i}},$$

5 $$H=\frac{P}{A},$$

where *P* is the peak load, *A* is the projected area at the peak load calculated from an empirical formula, *E*_*r*_ is the reduced elastic modulus, *E*_*i*_ is the elastic modulus, and *v*_*i*_ is Poisson’s ratio of the tips. For diamond tips, *E*_*i*_ is 1141 GPa, and *v*_*i*_ is 0.07. *E* and *v* are respectively the elastic modulus and Poisson’s ratio of the specimens^37^.

## Results and Discussion

### Microstructure

**Figure 4:**
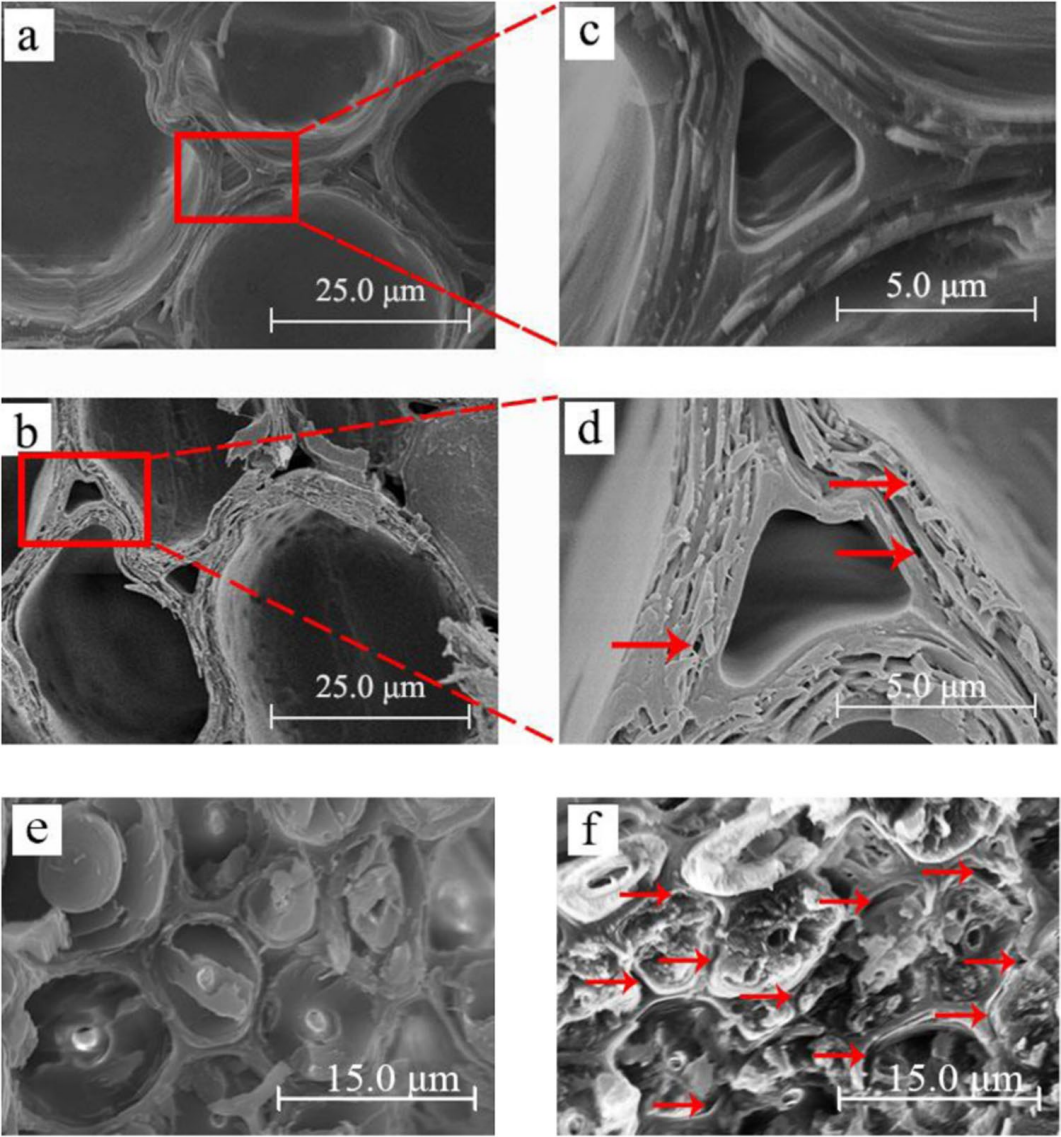
SEM images of bamboo cell wall structures after different hygrothermal treatments: (**a,c**) untreated parenchyma cells, (**b,d**) 220 °C treated parenchyma cells, (**e**) untreated fiber cells, (**f**) 220 °C treated fiber cells.

The Moso bamboo samples were fractured by hand after nitrogen freezing instead of by a cutter. Figure 4 displays the images of the parenchyma cells and the fiber cells of the bamboo samples, from which it is evident that the bamboo cell walls are multilayered structures, and are much more complicated than the three-layer structures of wood cell walls^38,39^.

The cross-sections of the parenchyma cells of the specimens are depicted in Fig. 4(a–d). The sample hygrothermally treated at 220 °C appeared to be more brittle than the untreated bamboo. By comparing the enlarged images in Fig. 4(c,d), it can also be seen micro-cracks and micropores originated from the parenchyma cell walls of the samples hygrothermally treated at 220 °C (denoted by red arrows in Fig. 4(d)). This result is in agreement with previous research, which reported that the main reason for this was the presence of extractives deposited in the cell wall that degraded after hygrothermal treatment^40^. The differences observed in the structural changes were also related to the chemical changes that took place during the hygrothermal treatment. The treated bamboo was found to contain less hemicellulose and lignin as compared to untreated bamboo. Hemicelluloses degraded violently at 220 °C, and the loss of hemicelluloses led to the increase of pores. Another reason for the origin of the micro-cracks and micropores was lignin. Lybeer found that the bamboo parenchyma secondary cell wall is composed of different narrower layers with alternating lignin contents^41^. The micro-cracks and micropores in Fig. 4(b,d) developed and enlarged principally as a result of the degradation of hemicellulose and lignin, which were the fillers and binders of cellulose microfibrils in the various cell wall layers.

A separation between two adjacent bamboo fiber cell walls occurred after hygrothermal treatment, as can be observed by comparing Fig. 4(e,f). Laminated gaps became obvious among the thick and thin layers (especially the S1 layer) of the bamboo fibers after treatment at 220 °C (see red arrow in Fig. 4(f)). This phenomenon is mainly attributable to the differences in the microfibril orientation and content of chemical components among the alternating thick and thin layers. In addition, the stress change due to the hygrothermal conditions also caused differential shrinkage^40^.

### Nitrogen adsorption

#### Nitrogen adsorption isotherm

There are various pores of different diameters in Moso bamboo. The IUPAC classification divides pores into three categories: micropores (pore diameter <2 nm), mesopores (pore diameter between 2 nm and 50 nm), and macropores (pore diameter >50 nm)^42,43^. Moreover, nitrogen adsorption-desorption isotherms can be used to classify the pores according to the IUPAC classification. To obtain more information on porosity in the bamboo cell walls, the nitrogen sorption method, which has proven to be effective in detecting mesopores (2–50 nm), was employed.

**Figure 5:**
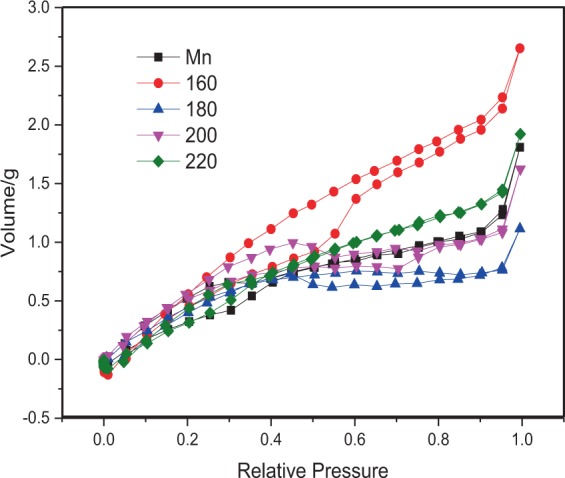
Nitrogen adsorption-desorption isotherms of Moso bamboo samples; Mn denotes control samples.

The nitrogen adsorption-desorption isotherms of the Moso bamboo samples are presented in Fig. 5. Referring to the IUPAC classification, the nitrogen adsorption-desorption isotherms of the bamboo were intermediate, between type II and type IV of the N_2_ absorption (BET specific surface area) isotherm type, and contained an H_3_ type of hysteresis loop^44^. Bamboo samples, in general, contain a large number of slit-shaped mesopores (diameters between 2 and 50 nm), as demonstrated by previous studies^37,45,46^.

#### Porosity

Table 1 provides the pore characteristics as determined by the N_2_ absorption (BET specific surface area) method. The specific surface area (*S*_*BET*_), total pore volume (*V*_*total*_), and the average mesopore diameter (*D*_*BJH*_) of the bamboo cell walls changed significantly after hygrothermal treatment.

**Table 1:** Pore characteristics of the Moso bamboo cell walls subjected to hygrothermal treatment.

	Mn	160 °C	180 °C	200 °C	220 °C
Sample Weight (g)	0.0903	0.0678	0.1093	0.1257	0.0749
*S*_*BET*_^a^ (m^2^·g^-1^)	2.32	2.93	2.54	2.63	3.41
*V*_*total*_^b^ (cm^3^·g^-1^)	2.80*10^-3^	4.10*10^-3^	1.73*10^-3^	2.51*10^-3^	2.97*10^-3^
*D*_*BJH*_^c^ (nm)	4.83	5.61	2.72	3.82	3.49

Note: M_n_ denotes control samples.

^a^The BET specific surface area. ^b^The total pore volume. ^c^The average mesopore diameter as determined via the BJH method.

It is evident from Table 1 that the *S*_*BET*_ values of the hygrothermally treated samples increased as compared to that of the untreated samples. The *S*_*BET*_ value reached a maximum at 220 °C, presenting an increase of 47% as compared to the control group. This phenomenon may be because the partial polysaccharides (especially hemicelluloses) and functional group of lignin degraded and hydrolyzed to hydrolysate, which dissolves easily in water during hygrothermal treatment^47^. Additionally, this result does not rule out the existence of interface damage between the fibers, which is caused by differential swelling stresses in the steam area.

*V*_*total*_ and *D*_*BJH*_ both reached their maximum values at 160 °C. The *V*_*total*_ value of the samples hygrothermally treated at 160 °C increased by nearly 46%, and the *D*_*BJH*_ value increased by 16%, as compared with the untreated samples. This is due to the degradation of hemicellulose and the release of some of the volatile organic compounds from the bamboo cell walls after the hygrothermal treatment at 160 °C, which significantly increased the total pore volume and the average pore size^48,49^. However, when the temperature was higher than 160 °C, the hemicellulose degradation was still processing, but the free spaces created by polyoses degradation could be refilled by the flowing chemical compositions in high-temperature conditions. Therefore, the average pore size of the samples decreased significantly. In addition, an increasing number of the hydrogen bonds within the celluloses also caused tighter cellulose chain arrangements^50^. Therefore, the total pore volume and average pore size decreased accordingly.

**Figure 6:**
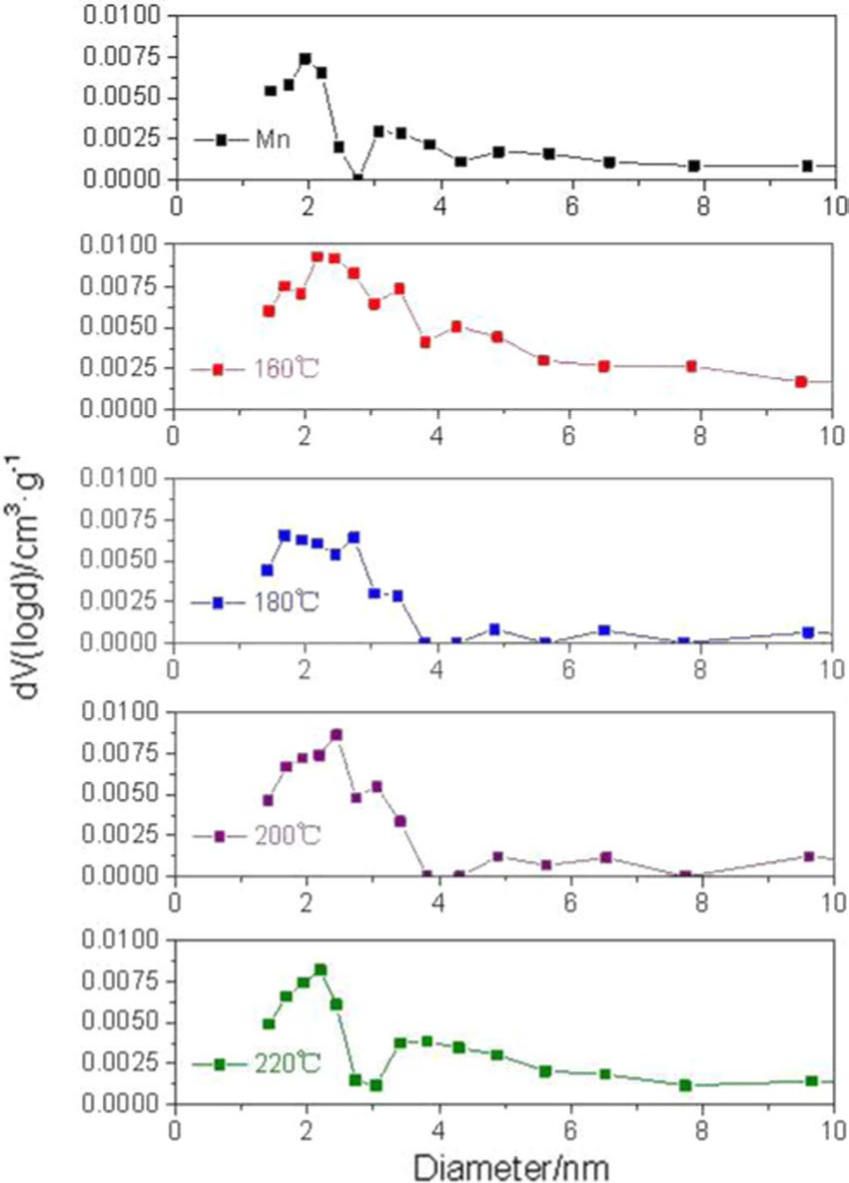
The mesopore size distributions of Moso bamboo at different conditions between 1.5 nm and 10 nm.

The specific surface area of the samples hygrothermally treated at 180 °C was found to be slightly larger than that of the untreated samples, but the *V*_*total*_ and *D*_*BJH*_ values decreased. This was mainly because of the differences between the bio-diversity characteristics of the bamboo sticks. Moreover, the *S*_*BET*_ area depends on the size of the micropore volume. The volumes of micropores are smaller than those of mesopores, but the specific surface areas of micropores are obviously larger than those of mesopores; the smaller pore diameter contributes to the larger specific surface area. However, in general, *D*_*BJH*_ = 4 * *V*_*total*_/*S*_*BET*_, which is in accordance with the BET rules. According to Fig. 6, the first peak of the 180 °C treated samples ranging from 1.8 to 2 nm presented a distinguished drop as compared to the those of untreated samples. Additionally, there were fewer mesopores of the 180 °C treated samples that were larger than 4 nm as compared to the control samples. Therefore, the *V*_*total*_ and *D*_*BJH*_ values both decreased.

#### Pores size distributions

Previous studies of pore structures in wood have explained that the micropores less than 2 nm in diameter are slit-shaped pores among the cell wall structures, while the mesopores in the range of 2–10 nm exist among the microfibrils and cell walls^51^. Mesopores larger than 10 nm are mainly the pits and other pores of the cell wall structures^52^, and are not discussed here.

Figure 6 exhibits the pore diameter distribution between 1.5 and 10 nm. For untreated bamboo, two peaks were observed at about 2 and 3 nm, respectively, indicating the frequent occurrence of mesopores of this diameter. Additionally, the first peak was significantly higher than the second, which means that there were more 2 nm pores than 3 nm pores. After hygrothermal treatment at 160 °C, the two peaks presented an obvious increase. The first peak became gentle, suggesting the increasing amount of mesopores within the diameter range of 1.5 nm to 3 nm during the 160 °C hygrothermal treatment. The peak at 3 nm shifted to the right, indicating the diameter enlargement of these mesopores. The increases of total pore volume and specific surface area also confirm the development in the pore size and amount caused by the degradation of hemicellulose and the volatilized extractive components after the hygrothermal treatment of 160 °C. As for the 180 °C treated samples, the first peak ranging from 1.8 nm to 2 nm had a distinguished drop as compared to the untreated samples. Lignin that moved or formed into pseudo-lignin under this condition probably clogged the pores (the pseudo-substances formed by the degradation products of lignin, hemicellulose, or even cellulose and adhere to the pores)^20,53^. The intensities of the two peaks at 2.3 nm and 3 nm strengthened in the 200 °C treatment, suggesting the increasing amount of the mesopores within this diameter range. A large number of mesopores ranging from 3.5 nm to 5 nm in diameter were found in the 220 °C hygrothermal treatment, which agrees with the increase of the average mesopore diameter and specific surface area as stated above. The diameter changes of these nano-pores are mainly due to the chemical changes in the amorphous zone between the microfilaments of the cell walls.

### Crystallinity and MFA

Table 2 shows the MFA and relative crystallinity of the hygrothermally-treated samples as observed by XRD. The relative crystallinity indices (CrI) first increased during the elevation of temperature, and reached a maximum (36.92%) at 180 °C. This was an increase of approximately 11.1% as compared to the control samples (33.24%).

**Table 2:** Crystallinity and MFA of Moso bamboo treated at different temperatures.

	Mn	160 °C	180 °C	200 °C	220 °C
CrI (%)	33.24	33.95	36.92	34.03	31.83
MFA (°)	11.66	11.41	11.29	11.26	11.16

Note: M_n_ denotes control samples.

Yun *et al*. (2016) found that the relative crystallinity of Moso bamboo fibers increased with the increase of temperature, exhibiting an increase of 5.67% after heat treatment at 180 °C as compared with the control samples^20^. Ma *et al*. (2011) also reported that the crystallinity index of bamboo cellulose increased after hydrothermal pretreatment at 180 °C^54^. Tanahashi *et al*. (1989) argued that the steaming of wood results in the increase of cellulose crystallinity and microfibril width, which is in agreement with the results of the present study. The increase in crystallinity found in the present study can be explained by the higher reactivity of the cellulose in the amorphous region as compared to that in the crystalline region. The hydroxyls of the amorphous region lost water through condensation and produced ether bonds at certain temperatures, which caused an increase in crystallinity^50,54,55^. On the other hand, the temperature condition exceeded the hemicellulose softening temperature, thus leading to the rearrangement of adjacent cellulose chains in the amorphous region and the formation of hydrogen bonds, which drove the microfibrils towards higher crystalline perfection^52^. In addition, the removal of the branched hemicellulose also increased the crystallinity index of the cellulose^56,57^. However, when the hygrothermal temperature reached 200 °C or even 220 °C, the relative crystallinity gradually decreased. This is because parts of the cellulose macromolecular chains in the crystalline region of the bamboo cell walls were degraded, and the tight structures were broken.

When the hygrothermal treatment temperature increased to 220 °C, MFA declined by nearly 4.5% as compared to the untreated samples. According to the study by Yun *et al*. (2016), the length-width ratio of bamboo fibers was increased by 36.9% after heat treatment at 180 °C^20^. As Parameswaran and Liese (1976) found, the microfibrils of the bamboo secondary cell wall structure are arranged in alternating axial and transverse directions, while axial micro fibrils account for greater a proportion in the multilayer structure. The reduction of MFA during hygrothermal treatment was probably a result of the steam softening of the lignin-hemicellulose matrix, which embedded in the crystalline cellulose microfibrils. The cellulose chains in the amorphous regions moved when the hemicellulose and some of the polymers degraded. This caused the partial reorientation of the cellulose microfibrils, resulting in the decrease of microfibril angles. After hygrothermal treatment, the changes of the samples were set^58^. Therefore, the rearrangement process resulted in the increase of the axial size of fibers, and the MFA consequently decreased.

### Nanoindentation

Table 3 presents the changes in the nanomechanical properties of the hygrothermally-treated bamboo samples as observed by nanoindentation.\

**Table 3:** Changes of physical and nanomechanical properties of the bamboo before and after hygrothermal treatment.

T (°C)	Time (h)	RH (%)	E (GPa)	Hardness (MPa)
**Mn**	—	—	17.83	489.09
**180**	1.5	117	21.42	591.03
**220**	1.5	117	20.49	579.91

Note: M_n_ denotes control samples.

**Figure 7:**
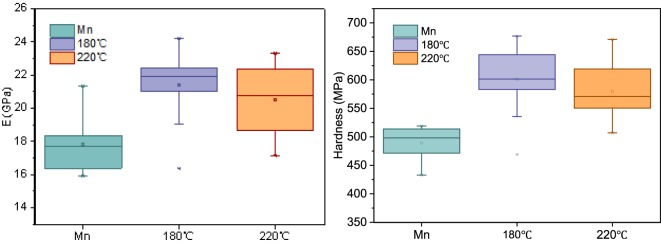
The changes of *E* and the hardness of bamboo cell walls after different hygrothermal treatments.

The average *E* and hardness values of the bamboo cell walls are listed in Table 3. These two values at 180 °C were found to be significantly higher than those of the untreated samples. The *E* and hardness values attained respective maximums of 21.42 GPa and 591.03 MPa at 180 °C, increasing almost 21% as compared with the control samples. When the hygrothermal treatment temperature increased to 220 °C, the average *E* and hardness values exhibited slightly decreasing trends (Fig. 7).

The nanomechanical properties of the cell walls are generally influenced by crystallinity, moisture content (MC), chemical composition, density, and MFA^58–60^. The changes of relative crystallinity significantly influence the *E* and hardness. Salmén illustrated that the amorphous regions of cellulose affect the mechanics of the fiber in the cross-sectional direction, while the proportion of the crystalline regions are the main determinant of the longitudinal nanomechanical properties of the cell walls^60^. As discussed previously, the hemicellulose degraded and the crystallinity increased after treatment at 180 °C. The degradation of the hemicellulose during the hygrothermal treatment refers to the reduction of hydrogen bonding between glucomannan and the cellulose fibril surface, and the reduction of covalent bonds among hemicelluloses and less-condensed and condensed lignin^44^. In addition, the crystallinity increased and the MFA decreased slightly, resulting in an increase of *E*. However, when the treatment temperature increased to 220 °C, parts of the cellulose chains degraded, the tight structures were broken, and the relative crystallinity decreased as the tight structures were broken, which resulted in the decrease of *E*.

Additionally, the increase of relative crystallization was not the only reason for the increase in the hardness values after hygrothermal treatment. The hygrothermally-induced condensation of lignin and its cross-linking reactions with furfural (which arise from the thermal degradation of pentoses) are also essential factors that contribute to elevated hardness^61^.

**Figure 8:**
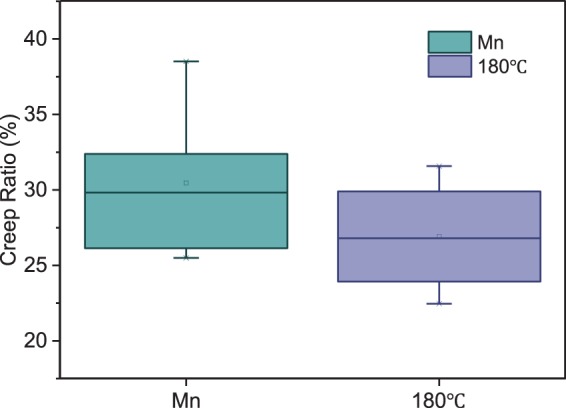
The change of the creep ratio of bamboo cell walls after hygrothermal treatment.

Excellent nanomechanical properties of the Moso bamboo cell walls were found after hygrothermal treatment at 180 °C. Therefore, creep testing was conducted to compare the control bamboo cell walls and the 180 °C hygrothermally-treated cell walls. Creep, which indicates the slow deformation of solid materials under the influence of stresses, should also occur during the NI. Figure 8 presents the creep ratios of the specimens. The indentation creep ratio of the control cell walls was 30.47%, which decreased to 26.91% after hygrothermal treatment at 180 °C and 117% RH. This reveals that hygrothermal treatment could increase the creep resistance and the nanomechanical properties of the cell walls of Moso bamboo. This phenomenon can be attributed to the increase of the crystallinity, as well as the formation of the cross-linkages between cell wall polymers, that occurred due to the hygrothermal treatment^61^. Moreover, hemicellulose, serving as a viscous matrix in microfibrils, was also a reason for the changes of the creep ratio. The hygrothermal degradation of hemicelluloses resulted in the weakening of the ductile connections between the cell wall polymers, and led to the decrease of the creep ratio. The relatively higher lignin content in the modified structure also significantly contributed to this effect^62^.

### Cell wall structure in response to hygrothermal treatment

**Figure 9:**
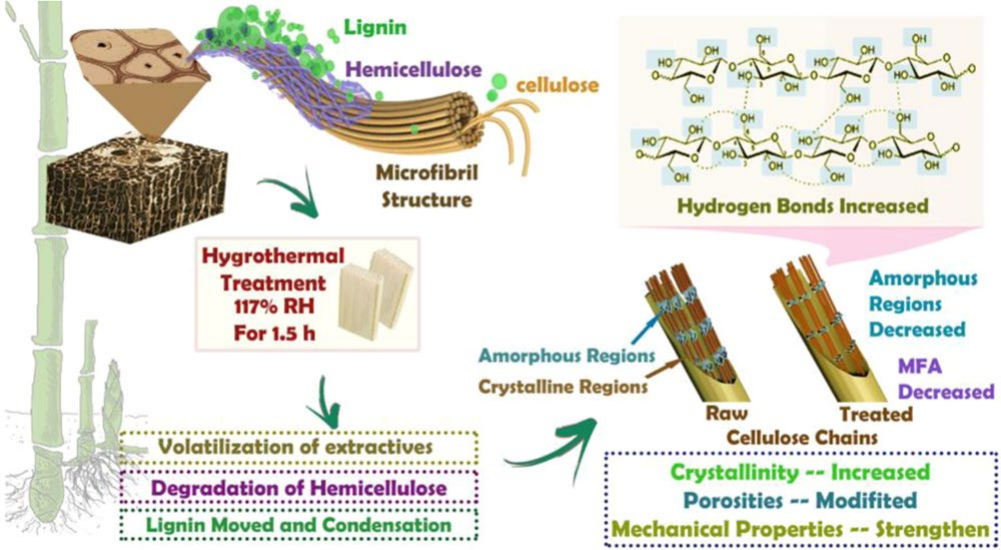
The mechanisms of the effects of hygrothermal treatment on bamboo cell walls.

The solid fraction of bamboo cell walls is filled with cellulose, lignin, hemicellulose, and extractive, and also exhibits a porosity of molecular-scale dimensions. It is believed that the physical, chemical, and nanomechanical properties of bamboo are governed by the complex arrangement of the solid fraction and the porosity of bamboo cell walls. As illustrated in Fig. 9, the release of volatile organic compounds, the degradation of hemicellulose, and the movement and condensation of lignin change the structure of bamboo cell walls, especially during hygrothermal treatment at and above 180 °C.

When hygrothermally-treated at 180 °C, a large portion of the hemicelluloses in the bamboo cell walls decomposed, and the esterification of the accessible hemicelluloses in the cell walls reduced hydrogen bonding with water^62^. Additionally, hygrothermal treatment caused the tight assembly of the cellulose chains and the increase of the relative crystallinity^50^. Therefore, the treated Moso bamboo became dimensionally more stable.

Many mesopores were formed during the hygrothermal treatment due to the degradation of amorphous polysaccharides (hemicellulose and lignin) and the volatilization of extractives, which led to a lower cell wall density^42^. However, after hygrothermal treatment at 180 °C, the total pore volume and the average pore size of the bamboo cell walls were low, which may have been due to the lignin that moved and blocked some mesoporous pores. Moreover, the increase of the relative crystallinity was also a result.

The nanomechanical properties of the cell walls are probably related to the crystallinity, the density, and the condensation of lignin. When the crystallinity of the cell walls increases at temperatures below 180 °C, the microfibrils in the amorphous region are arranged more regularly. Therefore, they require more stress to undergo deformation, which indicates increases of *E* and hardness. The decrease of the total pore volume and the average pore size of the bamboo cell walls also resulted in an improvement in *E* and hardness.

## Conclusion

Hygrothermal treatment is a preferable modification method for Moso bamboo, and causes obvious changes in crystallinity, porosity, and nanomechanical properties. The mechanical properties of the Moso bamboo cell walls are mainly affected by their relative crystallinity, moisture content (MC), and chemical compositions. Many mesopores were formed in the hygrothermally-treated bamboo cell walls, and the specific surface areas of the treated samples increased. However, the total pore volume and average pore diameter (2.72 nm) of the bamboo cell walls decreased to the lowest values during hygrothermal treatment at 180 °C and 117% RH for 1.5 h. Moreover, the relative crystallinity reached the maximum (36.92%) at this condition, exhibiting an increase of 11.07% as compared with the control samples. This is attributed to the degradation of hemicelluloses and the rearrangement of cellulose that occurred in the amorphous regions. Additionally, the number of -OH groups declined due to the hygrothermal treatment, resulting in the decrease of the moisture content of the bamboo cell walls. Lignin also moved and condensed at this period. These results all provide a better understanding of the excellent improvement of the nanomechanical properties of bamboo cell walls after hygrothermal treatment. The *E* and hardness of values of the bamboo cell walls hygrothermally treated at 180 °C both increased by nearly 21%, and the creep rate decreased by nearly 11.7%. Therefore, the hygrothermal treatment at 180 °C and 117% RH for 1.5 h was effective and enhanced the properties of Moso bamboo, and provides practical guidance for the high value-added utilization of modified Moso bamboo.
