# Author Correction: Effect of Hygrothermal Treatment on the Porous Structure and Nanomechanics of Moso Bamboo

**DOI:** 10.1038/s41598-020-75066-w

**Published:** 2020-10-20

**Authors:** Cuiyin Ye, Yanhui Huang, Qiming Feng, Benhua Fei

**Affiliations:** 1 Key Laboratory of Wooden Material Science and Application, Ministry of Education, Beijing Forestry University, Haidian, Beijing, 100083 People’s Republic of China; 2 International Center for Bamboo and Rattan, Beijing, 100102 People’s Republic of China

Correction to: *Scientific Reports* 10.1038/s41598-020-63524-4, published online 16 April 2020

The original version of this Article contained a repeated typographical error where,

“117% RH”

now reads:

“100% RH”

In addition, in Figure 9 the Hygrothermal Treatment was incorrectly labelled as “117% RH”, and now reads “100% RH”.

These errors have now been corrected in the HTML and PDF versions of the Article.

